# Supplementary material for: Patterns of Intron Gain and Loss in Fungi
Source: PLoS Biol. 2004 Nov 30;2(12):e422. doi: 10.1371/journal.pbio.0020422 (PMC532390; doi:10.1371/journal.pbio.0020422)
Supplement: Table S1 — Also available at http://genes.mit.edu/NielsenEtAl/. (4.3 MB ZIP). [file pbio.0020422.st001.zip › NielsenEtAl/html/987.html]

AN2105.1.NCU00505.1.MG04741.1.FG00358.1


```
 CLUSTAL W (1.82) Multiple Sequence Alignments - Introns Inserted


Sequence 1: AN2105.1	121 aa
Sequence 2: FG00358.1	126 aa
Sequence 3: NCU00505.1	123 aa
Sequence 4: MG04741.1	125 aa
Alignment Length: 128 aa
Number Identitical Residues: 89 aa
Alignment Score (without introns) 3554


MG04741.1 	MKLVR2FLMKCANETVTIELKNG1TIVHGTIASVSPQMNTALRNVKMTPRGQDPIPLDAM
NCU00505.1	MKLVR2FLMKCANETVTIELKNG~TIVHGTIASVTPRMDTALRNVKMTPKGQDAVALDAM
FG00358.1 	MKLVR2FLMKCANETVTIELKNG1TIIHGTISSVSPQMNTALRNVKMTIKGQEPISLETM
AN2105.1  	MKLVR2FLMKCANETVTIELKNG1TILHGTITSVSPQMNTSLRTVKMTPKGRDPISLDTI
          	***** ***************** **:****:**:*:*:*:**.**** :*::.:.*:::

MG04741.1 	NIR~GSTIRYYILPDSLPLDTLLVDDTPKPKNKARKETDRGGRGGGRGRGG-RGRG-GFG
NCU00505.1	NIR2GSTIRYFILPDSLPLDTLLVDDSVKPKNKARKEADRSAGGRG-GRGG-RGRGRGMG
FG00358.1 	NIR~GSTIRYFILPDSLPLDTLLIDDAPKPKNKARKEAADRGARGGRGRGGPRGRGRGGG
AN2105.1  	NIR~GSTIRYYILPDSLPLDTLLIDDTPKPKNKARKEA-DRG-RGG-GRGGPRGRGRGRG
          	*** ******:************:**: *********:   .   * ****.**** * *

MG04741.1 	RGRGFGRGRG-
NCU00505.1	RGR--GRGRG-
FG00358.1 	GGR--GRGGRP
AN2105.1  	RGR--GRGF--
          	 **  ***
```
